# Supplementary material for: Effect of Smoking on Treatment Efficacy and Toxicity in Patients with Cancer: A Systematic Review and Meta-Analysis
Source: Cancers (Basel). 2022 Aug 25;14(17):4117. doi: 10.3390/cancers14174117 (PMC9454993; doi:10.3390/cancers14174117)
Supplement: Supplementary file 1 [file cancers-14-04117-s001.zip › cancers-1827304-supplementary.pdf]

Supplementary Materials

Data synthesis

If standard errors (to enable weighting the effect sizes using the inverse variance method) were not provided directly, they were estimated from reported confidence intervals (see Supplementary material for detailes):

$$\widehat{se}_{log} = \frac{log\{\hat{\theta}_U\} - log\{\hat{\theta}_L\}}{2 \times 1.96} \qquad \text{Eq.1}$$

where $\hat{\theta}_U$ and $\hat{\theta}_L$ denote the upper- and lower- bound estimates of the effect size metric (i.e. HR or OR). Where only the effect size and *P* value were reported, the corresponding standard score (*z*) was derived from the standard normal distribution, which was then used to estimate the standard error using:

$$\widehat{se}_{log} = \frac{log\{\hat{\theta}\}}{z} \qquad \text{Eq.2}$$

where θ denotes either the HR or the OR.

Table S1. Characteristics of eligible studies

Author (Year of publication)	Type of study	Region	Cancer type	Treatment modality	N patients / N smokers	Outcome	Median F-up, in months	Inclusion in meta-analysis
Agarwal (2009)	Retrospective	Asia	Head & Neck cancer	Radiotherapy	627 / 505	LRR, DFS	11	Yes
Ajili (2013)	Retrospective	Africa	Bladder cancer	BCG immunotherapy	112 / 81	DFS	30	No
Alessi (2021)	Retrospective	N America	Lung cancer	Immunotherapy (pembrolizumab)	221 / 212	PFS	26.9	Yes
Al-Mamgani (2013)	Retrospective	Europe	Head & Neck cancer	Radiotherapy	906/700	LRR	90	Yes
Barnett (2011)	Retrospective	Europe	Breast cancer	Radiotherapy	1014 / NR	Toxicity (skin toxicity)	NR	Yes
Boeri (2019)	Retrospective	N America	Bladder cancer	Chemotherapy (cisplatin-based)	201/56	pCR, LRR	24.6	No
Cavallo (2021)	Retrospective	Europe	Head & neck cancer	Radiotherapy	132 / 24	Toxicity (salivary gland sysfunction)	NR	Yes
Cho (2019)	Retrospective	Asia	Lung cancer	Immunotherapy	178/111	ORR, PFS	NR	Yes
Choi (2014)	Retrospective	Asia	Lung cancer	TKIs-EGFR (gefitinib-erlotinib)	130/30	PFS	NR	Yes
Cortellini (2020)	Retrospective	Europe	Lung cancer	Immunotherapy	1026 / 348	ORR, PFS	14.6	Yes
Cozzarini (2016)	Prospective	Europe	Prostate cancer	Radiotherapy	125 / 22	Toxicity (lymphopenia)	NR	Yes
de Graan (2012)	Retrospective	Europe	Various cancer types	Chemotherapy (taxane)	276 / 62	Toxicity (neutropenia)	NR	Yes
de Jongh (2003)	Retrospective	Europe	Various cancer types	Chemotherapy (cisplatin)	400 / NR	Toxicity (renal, neuro-toxicity, ototoxicity)	NR	Yes
De Langhe (2014)	Retrospective	Europe	Breast cancer	Radiotherapy	374 / 51	Toxicity	NR	Yes
Dolan (2017)	Retrospective	N America	Germ cell tumours	Chemotherapy (cisplatin)	680 / 51	Toxicity (neurotoxicity)	NR	Yes
Engvall (2021)	Retrospective	Europe	Breast cancer	Chemotherapy (taxanes)	397 / 64	Toxicity (neurotoxicity)	NR	Yes
Fang (2018)	Retrospective	N America	Oesophageal cancer	CRT	313 / 66	pCR	NR	No

Fodor (2022)	Retrospective	Europe	Breast cancer	Radiotherapy	1325 / 263	Toxicity (skin)	72.4	Yes
Fortin (2008)	Retrospective	N America	Head & Neck cancer	Radiotherapy	1871 / 951	LRR	38	Yes
Fujimoto (2018)	Retrospective	Asia	Lung cancer	Immunotherapy (nivolumab)	613 / 482	PFS	NR	Yes
Fukuokaya (2021)	Retrospective	Asia	Bladder cancer	Immunotherapy (pembrolizumab)	59 / 19	PFS	8.2	Yes
Fung (2021)	Retrospective	N America	Head & neck cancer	CRT	334 / 169	DFS	NR	Yes
Furrer (2017)	Retrospective	N America	Breast cancer	Trastuzumab	131 / 17	DFS	88.8	No
Fyles (2002)	Prospective	N America	Cervical cancer	Radiotherapy	100 / 34	LRR, DFS	36	Yes
Gainor (2020)	Retrospective	N America	Lung cancer	Immunotherapy (any checkpoint inhibitor)	273/237	PFS	14.9	Yes
Gillison (2012)	Randomized	N America	Head & Neck cancer	Radiotherapy, CRT	256 / 23	DFS	111.6	Yes
Goldvaser (2017)	Retrospective	Asia	Breast cancer	Hormonal therapy, chemotherapy	617 / 101	DFS	61.9	No
Guo (2014)	Retrospective	Asia	Head & Neck cancer	CRT	400 / 193	DFS	80.2	Yes
Hoff (2012)	Prospective	Europe	Head & Neck cancer	Radiotherapy	232 / 162	LRR, DFS	NR	Yes
Huang (2014)	Retrospective	Asia	Oesophageal cancer	CRT	282 / 254	pCR	NR	No
Igawa (2016)	Retrospective	Asia	Lung cancer	Chemotherapy (pemetrexed)	116 / 78	PFS	12.9 (mean)	Yes
Isohashi (2012)	Retrospective	Asia	Cervical cancer	CRT	97 / 23	Toxicity (gastrointesti-nal)	43	No
Itaya (2007)	Retrospective	Asia	Lung cancer	Chemotherapy (carboplatin paclitaxel)	98 / 68	PFS	24.8	Yes
Ito (2021)	Retrospective	Asia	Lung cancer	Immunotherapy (PD-1 or PD-L1 inhibitors)	676 / 574	PFS	34.3	Yes
Jain (2015)	Retrospective	Asia	Lung cancer	TKIs EGFR<br>(gefitinib, erlotinib)	211 / 13	PFS	NR	Yes
Kanai (2009)	Retrospective	Asia	Pancreatic cancer	Chemotherapy (Gemcitabine)	103 / 51	Toxicity (neutropenia)	NR	Yes
Keizman (2013)	Retrospective	Asia, N	Renal cell carcinoma	TKIs (sunitinib)	278 / 59	PFS	55	No

		America						
Kelemen (2015)	Retrospective	N America	Ovarian cancer	Chemotherapy (mostly platina-based)	432 / 86	PFS	41.3	Yes
Kim (2013)	Retrospective	N America	Bladder cancer	Chemotherapy (cisplatin-based)	139 / 41	pCR, LRR	46	No
Kim (2014)	Retrospective	Asia	Lung cancer	TKIs EGFR (gefitinib, erlotinib)	222 / 77	PFS	33.6	Yes
Kim (2018)	Retrospective	Asia	Lung cancer	TKIs EGFR (gefitinib, erlotinib, afatinib, osimertinib)	142 / 51	PFS	19.8	Yes
Kim (2014)	Retrospective	Asia	Lung cancer	TKIs EGFR (gefitinib, erlotinib)	222 / 77	PFS	NR	No
Lan (2021)	Retrospective	Asia	Esophageal cancer	CRT	321 / 218	Toxicity (pneumonitis)	21.5	Yes
Levy (2014)	Retrospective	Europe	Head & Neck cancer	CRT (Cisplatin or cetuximab)	194 / 73	LRR, DFS	29	Yes
Lilla (2007)	Retrospective	Europe	Breast cancer	Radiotherapy	268 / 29	Toxicity (skin toxicity)	51	Yes
Lim (2014)	Retrospective	Asia	Lung cancer	TKIs EGFR (gefitinib, erlotinib)	242 / 88	PFS	NR	Yes
Liskamp (2016)	Retrospective	Europe	Head & neck cancer	CRT	142 / 65	LRR	33	Yes
Liu (2021)	Retrospective	Australia	Head & neck cancer	CRT	250 / 129	DFS	61	Yes
Li (2021)	Retrospective	Asia	Head & neck cancer	Radiotherapy	77 / 28	LRR	30.3	Yes
Lu (2013)	Retrospective	Asia	Lung cancer	Chemotherapy (pemetrexed)	119 / 68	ORR	NR	No
Luo (2022)	Retrospective	Asia	Lung cancer	Radiotherapy	111 / 79	LRR	NR	Yes
Mai (2007)	Retrospective	Europe	Anal cancer	RCT	68 / 34	LRR	22	Yes
Markoczy (2018)	Prospective	Europe	Lung cancer	TKIs EGFR (erlotinib)	62 / 20	PFS	13.4	Yes
Miao (2022)	Retrospective	Asia	Lung cancer	Immunotherapy (PD-1 or PD-L1 inhibitors)	351 / 227	PFS	NR	Yes
Mileshkin (2014)	Prospective	Australia	Endometrial cancer	CRT	346 / 98	DFS	NR	Yes
Miller (2019)	Retrospective	N America	Lung cancer	SBRT	203 / 55	DFS	21.9	Yes

Nakagawa (2012)	Prospective	Asia	Lung cancer	TKIs EGFR (erlotinib)	3439 / 1777	Toxicity (lung toxicity)	NR	No
Ng (2019)	Retrospective	Asia, N America	Lung cancer	Immunotherapy (PD1 or PDL1 in-hibitors)	189 / 97	PFS	7.1	Yes
Nilsson (2022)	Retrospective	Europe	Anal cancer	CRT	114 / 33	Toxicity (gastrointestinal	40	Yes
Nishinarita (2018)	Retrospective	Asia	Lung cancer	TKIs EGFR (gefitinib, erlotinib)	248 / 90	PFS	23.4	Yes
O´Malley (2013)	Retrospective	N America	Various cancer types	Chemotherapy (Gemcitabine)	137 / 12	Toxicity (neutropenia)	NR	Yes
Ouyang (2013)	Retrospective	Asia	Head & Neck cancer	Radiotherapy	1849 / 734	DFS	73.5	No
Pan (2014)	Retrospective	Asia	Lung cancer	TKIs EGFR (erlotinib, gefitinib)	48 / 11	PFS	NR	Yes
Pantarotto (2007)	Retrospective	N America	Prostate cancer	Radiotherapy	416 / 70	LRR, biochemical DFS	70.2	Yes
Park (2022)	Retrospective	Asia	Lung cancer	TKIs EGFR (osirmetinib)	61 / 22	PFS	36	Yes
Park (2021)	Retrospective	Asia	Head & neck cancer	Radiotherapy	103 / NR	Toxicity (dysgeusia)	NR	Yes
Park (2018)	Retrospective	Asia	Lung cancer	CRT	472 / 131	DFS	66.5	No
Park (2016)	Retrospective	Asia	Lung cancer	Chemotherapy (pemetrexed)	227 / 68	PFS	NR	Yes
Pasquier (2021)	Prospective	Europe	Breast cancer	Radiotherapy	288 / 79	Toxicity (skin)	25.2	Yes
Persson (2016)	Prospective	Europe	Breast cancer	Radiotherapy, endocrine treatment	1065 / 206	DFS	61	Yes
Pickles (2004)	Retrospective	N America	Prostate cancer	Radiotherapy	417 / 88	Biochemical DFS	59	No
Pignol (2014)	Prospective	N America, Europe	Breast cancer	Radiotherapy	257 / 25	Toxicity (skin toxicity)	NR	Yes
Sfakianos (2010)	Retrospective	N America	Bladder cancer	BCG	623 / 483	ORR, LRR, DFS	80.9	No
Sharp (2013)	Randomized	Europe	Breast cancer	Radiotherapy	384 / 32	Toxicity (skin toxicity)	NR	Yes

Sha (2021)	Retrospective	Asia	Lung cancer	Radiotherapy	126 / 55	Toxicity (pneumonitis)	NR	Yes
Simino (2020)	Prospective	S America	Various cancer types	Chemotherapy (various)	269 / 39	Toxicity (nausea)	NR	Yes
Solanki (2013)	Prospective	N America	Prostate cancer	Radiotherapy	633 / 124	biochemical DFS, toxicity (urogenital)	57	Yes
Spigel (2022)	RCT	Global	Lung cancer	Immunotherapy (durvalumab)	713 /649	PFS	34.2	Yes
Stankovic (2016)	Prospective	Europe	Prostate cancer	Radiotherapy	94 / 13	Toxicity (urogenital)	27	No
Steinberger (2015)	Retrospective	N America	Prostate cancer	Radiotherapy	2095 / 164	biochemical DFS, toxicity (urogenital)	95	Yes
Sunaga (2021)	Retrospective	Asia	Head & neck cancer	Radiotherapy	94 / 78	Toxicity (mucositis)	NR	Yes
Sun (2021)	Retrospective	Asia	Head & neck cancer	Radiotherapy	8649 / 2493	DFS	NR	Yes
Tenti (2022)	Retrospective	Europe	Prostate cancer	Radiotherapy	76 / 9	DFS	42.3	Yes
Tho (2005)	Retrospective	Europe	Breast cancer	Radiotherapy	92 / NR	Toxicity	NR	Yes
Trendowski (2021)	Retrospective	N America	Various	Chemotherapy	1045 / NR	Toxicity (neurotoxicity)	NR	Yes
Tsao (2006)	Retrospective	N America	Lung cancer	Chemotherapy (various)	873 / 355	PFS	40.7	Yes
Vatca (2014)	Retrospective	N America	Head & Neck cancer	CRT	72 / 30	Toxicity (mucositis)	26.4	No
Vilhonen (2019)	Retrospective	Europe	Lung cancer	Chemotherapy (various)	80 / 52	PFS	NR	Yes
Waggoner (2006)	Prospective	N America	Cervical cancer	CRT	316 / 133	DFS	NR	Yes
Wang (2021)	Retrospective	N America	Lung cancer	Immunotherapy (any checkpoint inhibitor)	269 / 1674	PFS	NR	Yes

Wang (2021)	Retrospective	Asia	Lung cancer	TKIs EGFR				
(icotinib)	2136 / 202	PFS	NR	Yes				
Won (2011)	Retrospective	Asia	Lung cancer	TKIs EGFR (gefitinib, erlotinib)	87 / 19	PFS	NR	Yes
Xie (2013)	Retrospective	Asia	Head & Neck cancer	Radiotherapy	75 / 37	DFS	25	No
Ying (2014)	Retrospective	Asia	Lung cancer	TKIs EGFR (gefitinib)	273 / 112	ORR, PFS	18.5	Yes
Zeng (2013)	Retrospective	Asia	Lung cancer	TKIs EGFR (gefitinib, erlotininb)	159 / 47	PFS	NR	Yes
Zhang (2022)	Retrospective	N America	Testicular cancer	Cisplatin	NR	Toxicity (neurotoxicity, tinnitus)	NR	Yes

Abbreviations: NR, not reported; BCG, Bacillus Calmette Guerin; TKIs, tyrosine kinase inhibitors; EGFR, Epidermal Growth Factor Receptor; CRT, chemoradiotherapy; LRR, locoregional recurrence rate; DFS, disease-free survival; pCR, pathological complete response; PFS, progression-free survival; ORR, objective response rate; NR, not reported.

Reference list of eligible studies

1. Agarwal JP, Mallick I, Bhutani R, et al. Prognostic factors in oropharyngeal cancer--analysis of 627 cases receiving definitive radiotherapy. Acta Oncol. 2009;48:1026-33.
2. Ajili F, Kourda N, Karay S, Darouiche A, Chebil M, Boubaker S. Impact of smoking intensity on outcomes of patients with non muscle invasive bladder cancer treated by BCG immunotherapy. Ultrastruct Pathol. 2013;37:273-7.
3. Alessi JV, Ricciuti B, Alden SL, et al. Low peripheral blood derived neutrophil-to-lymphocyte ratio (dNLR) is associated with increased tumor T-cell infiltration and favorable outcomes to first-line pembrolizumab in non-small cell lung cancer. J Immunother Cancer. 2021;9:e003536.
4. Al-Mamgani A, van Rooij PH, Woutersen DP, et al. Radiotherapy for T1-2N0 glottic cancer: a multivariate analysis of predictive factors for the long-term outcome in 1050 patients and a prospective assessment of quality of life and voice handicap index in a subset of 233 patients. Clin Otolaryngol. 2013;38:306-12.
5. Barnett GC, Wilkinson JS, Moody AM, et al. The Cambridge Breast Intensity-modulated Radiotherapy Trial: patient- and treatment-related factors that influence late toxicity. Clin Oncol (R Coll Radiol). 2011;23:662-73.
6. Boeri L, Soligo M, Frank I, et al. Cigarette smoking is associated with adverse pathological response and increased disease recurrence amongst patients with muscle-invasive bladder cancer treated with cisplatin-based neoadjuvant chemotherapy and radical cystectomy: a single-centre experience. BJU Int. 2019;123:1011-1019.
7. Cavallo A, Iacovelli NA, Facchinetti N, et al. Modelling Radiation-Induced Salivary Dysfunction during IMRT and Chemotherapy for Nasopharyngeal Cancer Patients. Cancers (Basel). 2021;13:3983.
8. Cho JH, Jung HA, Lee SH, et al. Impact of EGFR mutation on the clinical efficacy of PD-1 inhibitors in patients with pulmonary adenocarcinoma. J Cancer Res Clin Oncol. 2019;145:1341-1349.
9. Choi CM, Kim MY, Lee JC, Kim HJ. Advanced lung adenocarcinoma harboring a mutation of the epidermal growth factor receptor: CT findings after tyrosine kinase inhibitor therapy. Radiology. 2014;270:574-82.
10. Cortellini A, Tiseo M, Banna GL, et al. Clinicopathologic correlates of first-line pembrolizumab effectiveness in patients with advanced NSCLC and a PD-L1 expression of ⩾50. Cancer Immunol Immunother. 2020;69:2209-2221
11. Cozzarini C, Noris Chiorda B, Sini C, et al. Hematologic Toxicity in Patients Treated With Postprostatectomy Whole-Pelvis Irradiation With Different Intensity Modulated Radiation Therapy Techniques Is Not Negligible and Is Prolonged: Preliminary Results of a Longitudinal, Observational Study. Int J Radiat Oncol Biol Phys. 2016;95:690-5.
12. de Graan AJ, Loos WJ, Friberg LE, et al. Influence of smoking on the pharmacokinetics and toxicity profiles of taxane therapy. Clin Cancer Res. 2012;18:4425-32.
13. de Jongh FE, van Veen RN, Veltman SJ, et al. Weekly high-dose cisplatin is a feasible treatment option: analysis on prognostic factors for toxicity in 400 patients. Br J Cancer. 2003;88:1199-206.

14. De Langhe S, Mulliez T, Veldeman L, et al. Factors modifying the risk for developing acute skin toxicity after whole-breast intensity modulated radiotherapy. BMC Cancer. 2014;14:711.

15. Dolan ME, El Charif O, Wheeler HE, et al Platinum Study Group. Clinical and Genome-Wide Analysis of Cisplatin-Induced Peripheral Neuropathy in Survivors of Adult-Onset Cancer. Clin Cancer Res. 2017;23:5757-5768.

16. Engvall K, Gréen H, Fredriksson M, Åvall-Lundqvist E. Persistent neuropathy among early-stage breast cancer survivors in a population-based cohort. Br J Cancer. 2021;125:445-457.

17. Fang P, Jiang W, Davuluri R, et al. High lymphocyte count during neoadjuvant chemoradiotherapy is associated with improved pathologic complete response in esophageal cancer. Radiother Oncol. 2018;128:584-590.

18. Fodor A, Brombin C, Mangili P, et al. Toxicity of Hypofractionated Whole Breast Radiotherapy Without Boost and Timescale of Late Skin Responses in a Large Cohort of Early-Stage Breast Cancer Patients. Clin Breast Cancer. 2022;22:e480-e487.

19. Fortin A, Wang CS, Vigneault E. Influence of smoking and alcohol drinking behaviors on treatment outcomes of patients with squamous cell carcinomas of the head and neck. Int J Radiat Oncol Biol Phys. 2009;74:1062-9.

20. Fujimoto D, Yoshioka H, Kataoka Y, et al. Efficacy and safety of nivolumab in previously treated patients with non-small cell lung cancer: A multicenter retrospective cohort study. Lung Cancer. 2018;119:14-20.

21. Fukuokaya W, Kimura T, Yanagisawa T, Kimura S, Tsuzuki S, Koike Y, Iwamoto Y, Enei Y, Tanaka M, Urabe F, Onuma H, Honda M, Miki J, Oyama Y, Abe H, Egawa S. Impact of Dose-Effect in Smoking on the Effectiveness of Pembrolizumab in Patients with Metastatic Urothelial Carcinoma. Target Oncol. 2021;16:189-196.

22. Fung AS, Afzal AR, Banerjee R, Debenham B, Hao D. A real-world comparison of cisplatin vs cetuximab used concurrently with radiation in the treatment of locally advanced oropharyngeal carcinoma. Head Neck. 2021;43:153-163.

23. Furrer D, Jacob S, Michaud A, Provencher L, Lemieux J, Diorio C. Association of Tobacco Use, Alcohol Consumption and HER2 Polymorphisms With Response to Trastuzumab in HER2-Positive Breast Cancer Patients. Clin Breast Cancer. 2018;18:e687-e694.

24. Fyles A, Voduc D, Syed A, Milosevic M, Pintilie M, Hill R. The effect of smoking on tumour oxygenation and treatment outcome in cervical cancer. Clin Oncol (R Coll Radiol). 2002;14:442-6.

25. Gainor JF, Rizvi H, Jimenez Aguilar E, et al. Clinical activity of programmed cell death 1 (PD-1) blockade in never, light, and heavy smokers with non-small-cell lung cancer and PD-L1 expression ⩾50. Ann Oncol. 2020;31:404-411.

26. Gillison ML, Zhang Q, Jordan R, et al. Tobacco smoking and increased risk of death and progression for patients with p16-positive and p16-negative oropharyngeal cancer. J Clin Oncol. 2012;30:2102-11.

27. Goldvaser H, Gal O, Rizel S, et al. The association between smoking and breast cancer characteristics and outcome. BMC Cancer. 2017;17:624.

28. Guo SS, Huang PY, Chen QY, et al. The impact of smoking on the clinical outcome of locoregionally advanced nasopharyngeal carcinoma after chemoradiotherapy. Radiat Oncol. 2014;9:246.

29. Hoff CM, Grau C, Overgaard J. Effect of smoking on oxygen delivery and outcome in patients treated with radiotherapy for head and neck squamous cell carcinoma--a prospective study. Radiother Oncol. 2012;103:38-44.

30. Huang RW, Chao YK, Wen YW, et al. Predictors of pathological complete response to neoadjuvant chemoradiotherapy for esophageal squamous cell carcinoma. World J Surg Oncol. 2014;12:170

31. Igawa S, Sasaki J, Otani S, et al. Smoking History as a Predictor of Pemetrexed Monotherapy in Patients with Non-Squamous Non-Small Cell Lung Cancer. Oncology. 2016;91:41-7.

32. Isohashi F, Yoshioka Y, Mabuchi S, et al. Dose-volume histogram predictors of chronic gastrointestinal complications after radical hysterectomy and postoperative concurrent nedaplatin-based chemoradiation therapy for early-stage cervical cancer. Int J Radiat Oncol Biol Phys. 2013;85:728-34.

33. Itaya T, Yamaoto N, Ando M, et al. Influence of histological type, smoking history and chemotherapy on survival after first-line therapy in patients with advanced non-small cell lung cancer. Cancer Sci. 2007;98:226-30.

34. Ito S, Asahina H, Honjo O, et al; Hokkaido Lung Cancer Clinical Study Group Trial. Prognostic factors in patients with advanced non-small cell lung cancer after long-term Anti-PD-1 therapy (HOT1902). Lung Cancer. 2021;156:12-19.

35. Jain A, Lim C, Gan EM, et al. Impact of Smoking and Brain Metastasis on Outcomes of Advanced EGFR Mutation Lung Adenocarcinoma Patients Treated with First Line Epidermal Growth Factor Receptor Tyrosine Kinase Inhibitors. PLoS One. 2015;10:e0123587.

36. Kanai M, Morita S, Matsumoto S, et al. A history of smoking is inversely correlated with the incidence of gemcitabine-induced neutropenia. Ann Oncol. 2009;20:1397-401.

37. Keizman D, Gottfried M, Ish-Shalom M, et al. Active smoking may negatively affect response rate, progression-free survival, and overall survival of patients with metastatic renal cell carcinoma treated with sunitinib. Oncologist. 2014;19:51-60.

38. Kelemen LE, Warren GW, Koziak JM, Köbel M, Steed H. Smoking may modify the association between neoadjuvant chemotherapy and survival from ovarian cancer. Gynecol Oncol. 2016;140:124-30.

39. Kim PH, Kent M, Zhao P, et al. The impact of smoking on pathologic response to neoadjuvant cisplatin-based chemotherapy in patients with muscle-invasive bladder cancer. World J Urol. 2014;32:453-9.

40. Kim MH, Kim HR, Cho BC, et al. Impact of cigarette smoking on response to epidermal growth factor receptor (EGFR)-tyrosine kinase inhibitors in lung adenocarcinoma with activating EGFR mutations. Lung Cancer. 2014;84:196-202.

41. Kim IA, Lee JS, Kim HJ, Kim WS, Lee KY. Cumulative smoking dose affects the clinical outcomes of EGFR-mutated lung adenocarcinoma patients treated with EGFR-TKIs: a retrospective study. BMC Cancer. 2018;18:768.

42. Kim HR, Lee JC, Kim YC, et al. Clinical characteristics of non-small cell lung cancer patients who experienced acquired resistance during gefitinib treatment. Lung Cancer. 2014;83:252-8.

43. Lan K, Xu C, Liu S, Zhu J, Yang Y, Zhang L, Guo S, Xi M. Modeling the risk of radiation pneumonitis in esophageal squamous cell carcinoma treated with definitive chemoradiotherapy. Esophagus. 2021;18:861-871.

44. Levy A, Blanchard P, Bellefqih S, et al. Concurrent use of cisplatin or cetuximab with definitive radiotherapy for locally advanced head and neck squamous cell carcinomas. Strahlenther Onkol. 2014;190:823-31.

45. Lilla C, Ambrosone CB, Kropp S, et al. Predictive factors for late normal tissue complications following radiotherapy for breast cancer. Breast Cancer Res Treat. 2007;106:143-50.

46. Lim SH, Lee JY, Sun JM, Ahn JS, Park K, Ahn MJ. Comparison of clinical outcomes following gefitinib and erlotinib treatment in non-small-cell lung cancer patients harboring an epidermal growth factor receptor mutation in either exon 19 or 21. J Thorac Oncol. 2014;9:506-11.

47. Liskamp CP, Janssens GO, Bussink J, Melchers WJ, Kaanders JH, Verhoef CG. Adverse effect of smoking on prognosis in human papillomavirus-associated oropharyngeal carcinoma. Head Neck. 2016;38:1780-1787.

48. Liu HY, Daniels CP, Trada Y, et al. The importance of smoking status at diagnosis in human papillomavirus-associated oropharyngeal cancer. Head Neck. 2021;43:1440-1450.

49. Li X, Kitpanit S, Lee A, Mah D, et al. Toxicity Profiles and Survival Outcomes Among Patients With Nonmetastatic Nasopharyngeal Carcinoma Treated With Intensity-Modulated Proton Therapy vs Intensity-Modulated Radiation Therapy. JAMA Netw Open. 2021;4:e2113205.

50. Lu YY, Huang XE, Xu L, et al. Potential predictors of sensitivity to pemetrexed as first-line chemotherapy for patients with advanced non-squamous NSCLCs. Asian Pac J Cancer Prev. 2013;14:2005-8.

51. Luo LM, Wang Y, Lin PX, Su CH, Huang BT. The Clinical Outcomes, Prognostic Factors and Nomogram Models for Primary Lung Cancer Patients Treated With Stereotactic Body Radiation Therapy. Front Oncol. 2022;12:863502.

52. Mai SK, Welzel G, Haegele V, Wenz F. The influence of smoking and other risk factors on the outcome after radiochemotherapy for anal cancer. Radiat Oncol. 2007;2:30.

53. Markóczy Z, Sárosi V, Kudaba I, et al. Erlotinib as single agent first line treatment in locally advanced or metastatic activating EGFR mutation-positive lung adenocarcinoma (CEETAC): an open-label, non-randomized, multicenter, phase IV clinical trial. BMC Cancer. 2018;18:598.

54. Miao K, Zhang X, Wang H, et al. Real-World Data of Different Immune Checkpoint Inhibitors for Non-Small Cell Lung Cancer in China. Front Oncol. 2022;12:859938.

55. Mileshkin L, Paramanathan A, Kondalsamy-Chennakesavan S, Bernshaw D, Khaw P, Narayan K. Smokers with cervix cancer have more uterine corpus invasive disease and an increased risk of recurrence after treatment with chemoradiation. Int J Gynecol Cancer. 2014;24:1286-91.

56. Miller CJ, Martin B, Stang K, et al. Predictors of Distant Failure After Stereotactic Body Radiation Therapy for Stages I to IIA Non-Small-Cell Lung Cancer. Clin Lung Cancer. 2019;20:37-42.

57. Nakagawa K, Kudoh S, Ohe Y, et al. Postmarketing surveillance study of erlotinib in Japanese patients with non-small-cell lung cancer (NSCLC): an interim analysis of 3488 patients (POLARSTAR). J Thorac Oncol. 2012;7:1296-303.

58. Ng TL, Liu Y, Dimou A, et al. Predictive value of oncogenic driver subtype, programmed death-1 ligand (PD-L1) score, and smoking status on the efficacy of PD-1/PD-L1 inhibitors in patients with oncogene-driven non-small cell lung cancer. Cancer. 2019;125:1038-1049.

59. Nilsson MP, Gunnlaugsson A, Johnsson A, Scherman J. Dosimetric and Clinical Predictors for Acute and Late Gastrointestinal Toxicity Following Chemoradiotherapy of Locally Advanced Anal Cancer. Clin Oncol (R Coll Radiol). 2022;34:e35-e44.

60. Nishinarita N, Igawa S, Kasajima M, et al. Smoking History as a Predictor of Epidermal Growth Factor Receptor Tyrosine Kinase Inhibitors in Patients with Non-Small Cell Lung Cancer Harboring EGFR Mutations. Oncology. 2018;95:109-115.

61. O'Malley M, Healy P, Daignault S, Ramnath N. Cigarette smoking and gemcitabine-induced neutropenia in advanced solid tumors. Oncology. 2013;85:216-22.

62. Ouyang PY, Su Z, Mao YP, et al. Prognostic impact of cigarette smoking on the survival of patients with established nasopharyngeal carcinoma. Cancer Epidemiol Biomarkers Prev. 2013;22:2285-94.

63. Pan JB, Hou YH, Zhang GJ. Correlation between efficacy of the EGFR tyrosine kinase inhibitor and serum tumor markers in lung adenocarcinoma patients. Clin Lab. 2014;60:1439-47.

64. Pantarotto J, Malone S, Dahrouge S, Gallant V, Eapen L. Smoking is associated with worse outcomes in patients with prostate cancer treated by radical radiotherapy. BJU Int. 2007;99:564-9.

65. Park JY, Jang SH, Lee CY, et al. Pretreatment Neutrophil-to-Lymphocyte Ratio and Smoking History as Prognostic Factors in Advanced Non-Small Cell Lung Cancer Patients Treated with Osimertinib. Tuberc Respir Dis (Seoul). 2022;85:155-164.

66. Park D, Jain S, Quay-De La Vallee Z, Huber K, O'Leary M, Farag AM. Influence of smoking history on the perception of radiation-induced dysgeusia/hypogeusia in patients with head and neck cancer. Eur Arch Otorhinolaryngol. 2021;278:2993-3001.

67. Park SE, Noh JM, Kim YJ, et al. EGFR Mutation Is Associated with Short Progression-Free Survival in Patients with Stage III Non-squamous Cell Lung Cancer Treated with Concurrent Chemoradiotherapy. Cancer Res Treat. 2019;51:493-501

68. Park S, Kim HJ, Choi CM, et al. Predictive factors for a long-term response duration in non-squamous cell lung cancer patients treated with pemetrexed. BMC Cancer. 2016;16:417.

69. Pasquier D, Bataille B, Le Tinier F, et al. Correlation between toxicity and dosimetric parameters for adjuvant intensity modulated radiation therapy of breast cancer: a prospective study. Sci Rep. 2021;11:3626.

70. Persson M, Simonsson M, Markkula A, Rose C, Ingvar C, Jernström H. Impacts of smoking on endocrine treatment response in a prospective breast cancer cohort. Br J Cancer. 2016;115:382-90.

71. Pickles T, Liu M, Berthelet E, Kim-Sing C, Kwan W, Tyldesley S; PROSTATE COHORT OUTCOMES INITIATIVE. The effect of smoking on outcome following external radiation for localized prostate cancer. J Urol. 2004;171:1543-6.

72. Pignol JP, Vu TT, Mitera G, Bosnic S, Verkooijen HM, Truong P. Prospective evaluation of severe skin toxicity and pain during postmastectomy radiation therapy. Int J Radiat Oncol Biol Phys. 2015;91:157-64.

73. Sfakianos JP, Shariat SF, Favaretto RL, Rioja J, Herr HW. Impact of smoking on outcomes after intravesical bacillus Calmette-Guérin therapy for urothelial carcinoma not invading muscle of the bladder. BJU Int. 2011;108:526-30

74. Sharp L, Johansson H, Hatschek T, Bergenmar M. Smoking as an independent risk factor for severe skin reactions due to adjuvant radiotherapy for breast cancer. Breast. 2013;22:634-8.

75. Sha S, Dong J, Wang M, Chen Z, Gao P. Risk factors for radiation-induced lung injury in patients with advanced non-small cell lung cancer: implication for treatment strategies. World J Surg Oncol. 2021;19:214.

76. Simino GPR, Reis IA, Acurcio FA, Andrade EIG, Brazil NML, Cherchiglia ML. Risk factors associated with antineoplastic chemotherapy-induced nausea and vomiting. Rev Saude Publica. 2020;54:106.

77. Solanki AA, Liauw SL. Tobacco use and external beam radiation therapy for prostate cancer: Influence on biochemical control and late toxicity. Cancer. 2013;119:2807-14.

78. Spigel DR, Faivre-Finn C, Gray JE, et al. Five-Year Survival Outcomes From the PACIFIC Trial: Durvalumab After Chemoradiotherapy in Stage III Non-Small-Cell Lung Cancer. J Clin Oncol. 2022;40:1301-1311.

79. Stankovic V, Džamic Z, Pekmezovic T, et al. Acute and Late Genitourinary Toxicity after 72 Gy of Conventionally Fractionated Conformal Radiotherapy for Localised Prostate Cancer: Impact of Individual and Clinical Parameters. Clin Oncol (R Coll Radiol). 2016;28:577-86.

80. Steinberger E, Kollmeier M, McBride S, Novak C, Pei X, Zelefsky MJ. Cigarette smoking during external beam radiation therapy for prostate cancer is associated with an increased risk of prostate cancer-specific mortality and treatment-related toxicity. BJU Int. 2015;116:596-603.

81. Sunaga T, Nagatani A, Fujii N, Hashimoto T, Watanabe T, Sasaki T. The association between cumulative radiation dose and the incidence of severe oral mucositis in head and neck cancers during radiotherapy. Cancer Rep (Hoboken). 2021;4:e1317.

82. Sun XS, Xie SY, Luo DH, et al. Impact of smoking on survival in nasopharyngeal carcinoma: A cohort study with 23,325 patients diagnosed from 1990 to 2016. Radiother Oncol. 2021;162:7-17.

83. Tenti MV, Ingrosso G, Bini V, et al. Tomotherapy-based moderate hypofractionation for localized prostate cancer: a mono-institutional analysis. Rep Pract Oncol Radiother. 2022;27:142-151.

84. Tho LM, McIntyre A, Rosst A, et al. Acute supraclavicular skin toxicity in patients undergoing radiotherapy for breast cancer: an evaluation of the 'T'-grip method of patient positioning. Clin Oncol (R Coll Radiol). 2006;18:133-8.

85. Trendowski MR, Lusk CM, Ruterbusch JJ, Seaton R, Simon MS, Greenwald MK, Harper FWK, Beebe-Dimmer JL, Schwartz AG. Chemotherapy-induced peripheral neuropathy in African American cancer survivors: Risk factors and quality of life outcomes. Cancer Med. 2021;10:8151-8161.

86. Tsao AS, Liu D, Lee JJ, Spitz M, Hong WK. Smoking affects treatment outcome in patients with advanced nonsmall cell lung cancer. Cancer. 2006;106:2428-36.

87. Vatca M, Lucas JT Jr, Laudadio J, et al. Retrospective analysis of the impact of HPV status and smoking on mucositis in patients with oropharyngeal squamous cell carcinoma treated with concurrent chemotherapy and radiotherapy. Oral Oncol. 2014;50:869-76.

88. Vilhonen H, Kurki S, Laitinen T, Hirsjärvi S. Retrospective Evaluation of Lung Adenocarcinoma Patients Progressing on 1st Line Chemotherapy. Medicina (Kaunas). 2019;55:743.

89. Waggoner SE, Darcy KM, Fuhrman B, et al; Gynecologic Oncology Group. Association between cigarette smoking and prognosis in locally advanced cervical carcinoma treated with chemoradiation: a Gynecologic Oncology Group study. Gynecol Oncol. 2006;103:853-8.

90. Wang X, Ricciuti B, Alessi JV, Nguyen T, Awad MM, Lin X, Johnson BE, Christiani DC. Smoking History as a Potential Predictor of Immune Checkpoint Inhibitor Efficacy in Metastatic Non-Small Cell Lung Cancer. J Natl Cancer Inst. 2021;113:1761–9.

91. Wang Y, Yuan X, Yang M, et al. Efficacy of Icotinib, an EGFR Tyrosine Kinase Inhibitor in Non-Small Cell Lung Cancer Patients with Exon 19 Deletion and Exon 21 L858R: A Retrospective Analysis in China. Pharmacology. 2021;106:658-666.

92. Won YW, Han JY, Lee GK, et al. Comparison of clinical outcome of patients with non-small-cell lung cancer harbouring epidermal growth factor receptor exon 19 or exon 21 mutations. J Clin Pathol. 2011;64:947-52.

93. Xie X, Jin H, Hu J, et al. Association between single nucleotide polymorphisms in the p53 pathway and response to radiotherapy in patients with nasopharyngeal carcinoma. Oncol Rep. 2014;31:223-31.

94. Ying H, Yang XD, Sun Z, et al. Lifestyle risks exposure and response predictor of gefitinib in patients with non-small cell lung cancer. Med Oncol. 2014;31:220.

95. Zeng Z, Chen HJ, Yan HH, Yang JJ, Zhang XC, Wu YL. Sensitivity to epidermal growth factor receptor tyrosine kinase inhibitors in males, smokers, and non-adenocarcinoma lung cancer in patients with EGFR mutations. Int J Biol Markers. 2013;28:249-58.

96. Zhang X, Trendowski MR, Wilkinson E, et al. Pharmacogenomics of cisplatin-induced neurotoxicities: Hearing loss, tinnitus, and peripheral sensory neuropathy. Cancer Med. 2022 Mar 23.

97. Zhang CC, Hou RP, Xia Wy, et al. Prognostic index for estimating the survival benefit of postoperative radiotherapy in pathologic N2 non-small cell lung cancer: A real-world validation study. Lung Cancer. 2021;156:100-108.

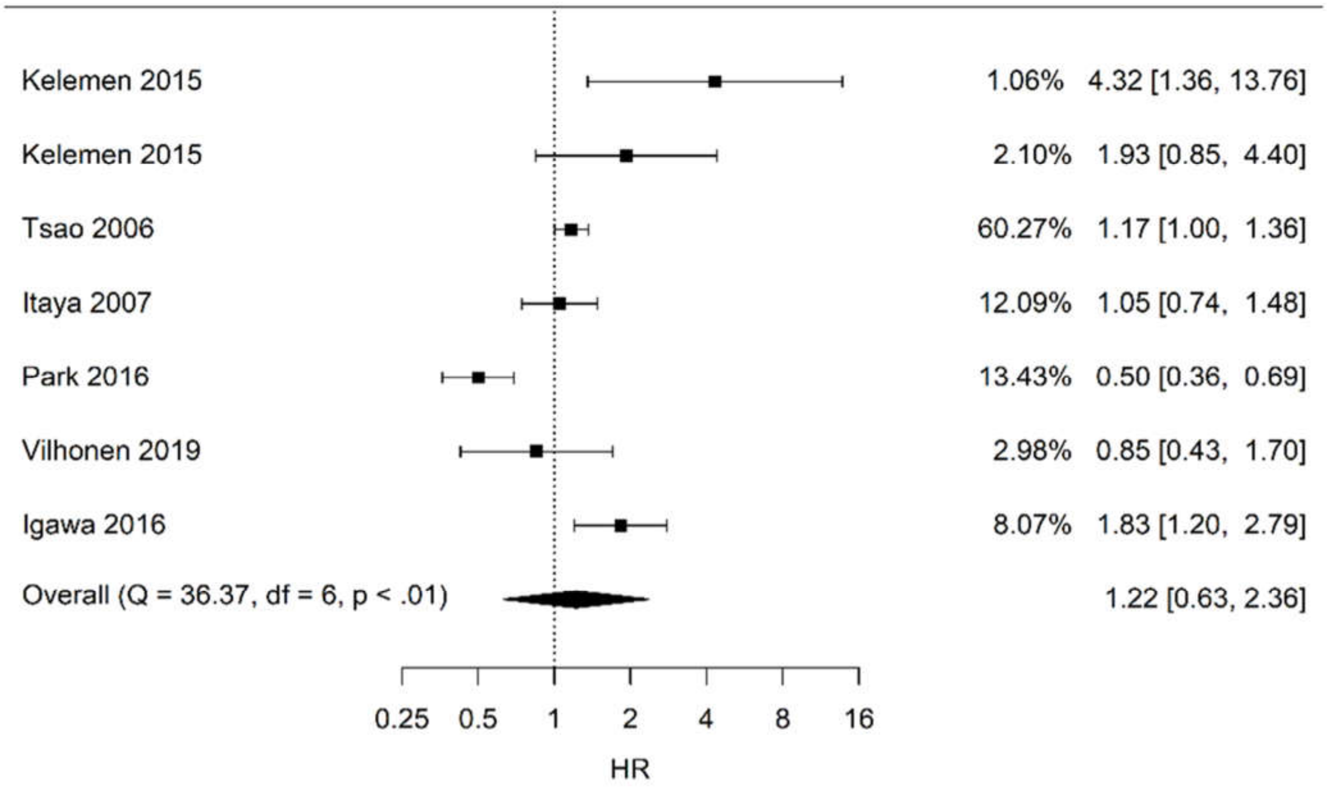

Figure S1 Forest plots on pooled Hazard Ratios (HR) on smoking during chemotherapy and efficacy in terms of progression-free survival (PFS)

HR > 1 indicates worse PFS for smokers during chemotherapy treatment while HR <1 indicates better PFS. Comparison group is non-smoker (former or never)

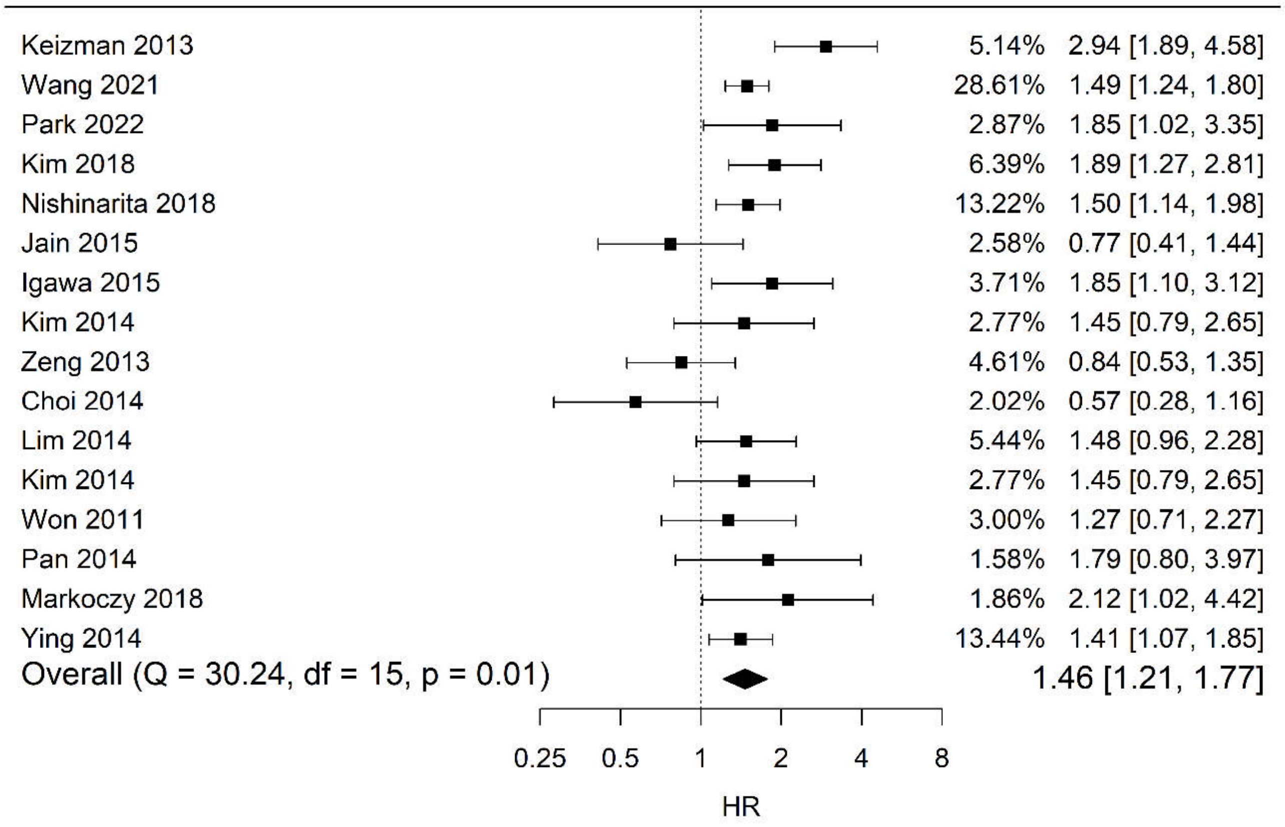

Figure S2 Forest plots on pooled Hazard Ratios (HR) on smoking during treatment with EGFR-tyrosine kinase inhibitors (EGFR-TKIs) and efficacy in terms of progression-free survival (PFS)

HR > 1 indicates worse PFS for smokers during EGFR-TKIs treatment while HR <1 indicates better PFS. Comparison group is non-smoker (former or never)

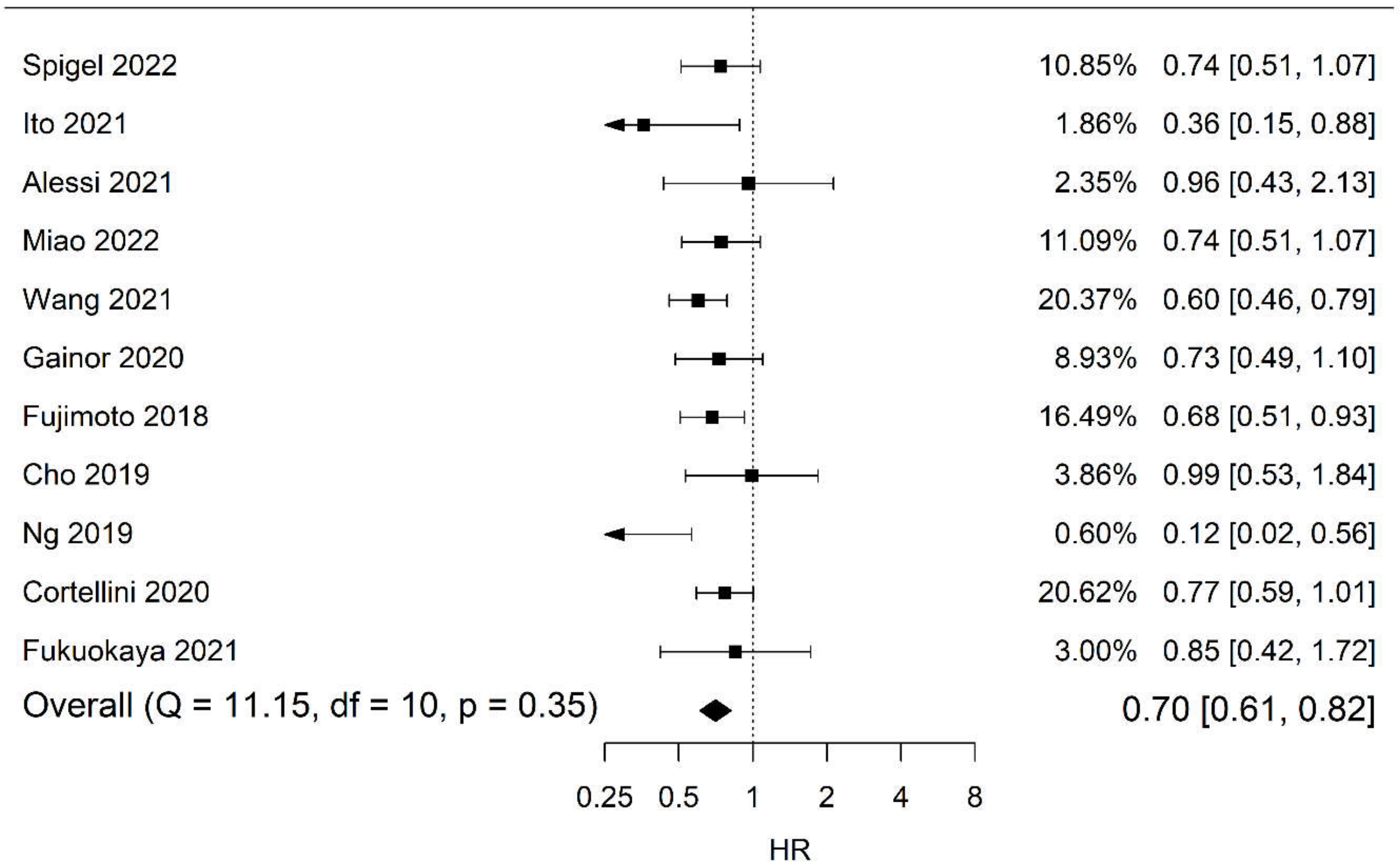

Figure S3 Forest plots on pooled Hazard Ratios (HR) on smoking during treatment with immunotherapy (checkpoint inhibitors) and efficacy in terms of progression-free survival (PFS)

HR > 1 indicates worse PFS for smokers during immunotherapy while HR <1 indicates better PFS. Comparison group is non-smoker (former or never)

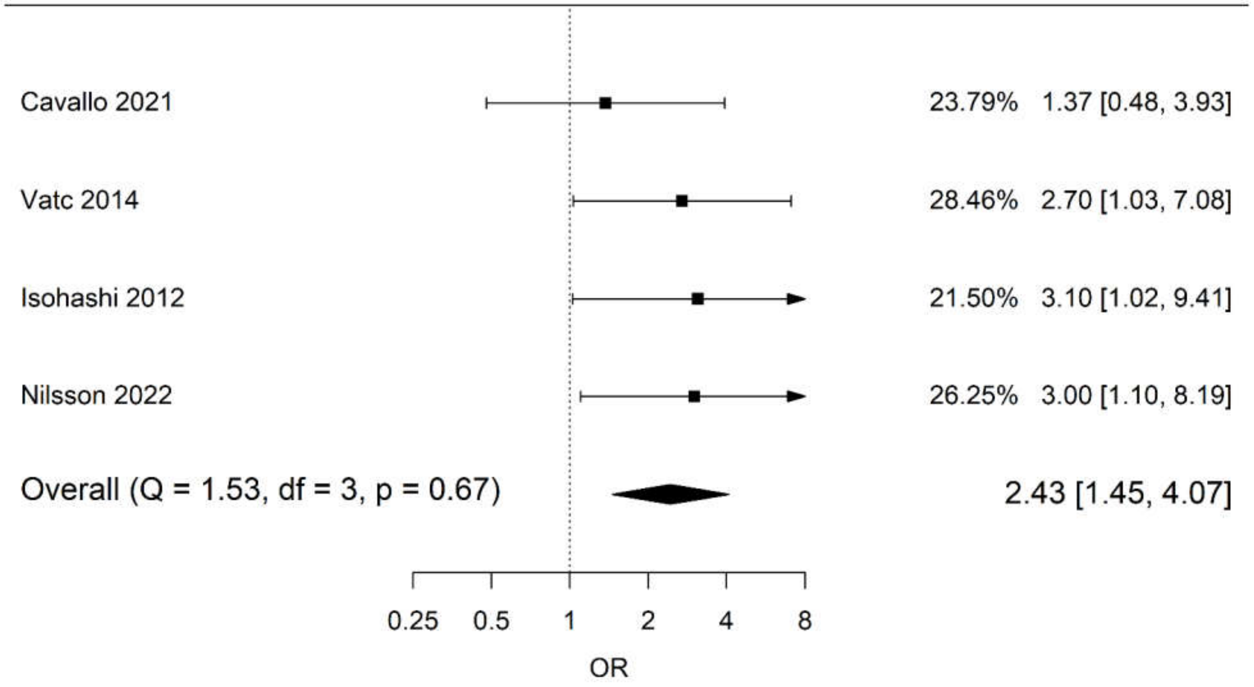

Figure S4. Forest plots on pooled Odds Ratios (OR) on smoking during radiochemotherapy and toxicity.

OR > 1 indicates higher risk for toxicity during treatment whereas OR < 1 indicates lower risk. Comparison group is non-smokers (former or never).

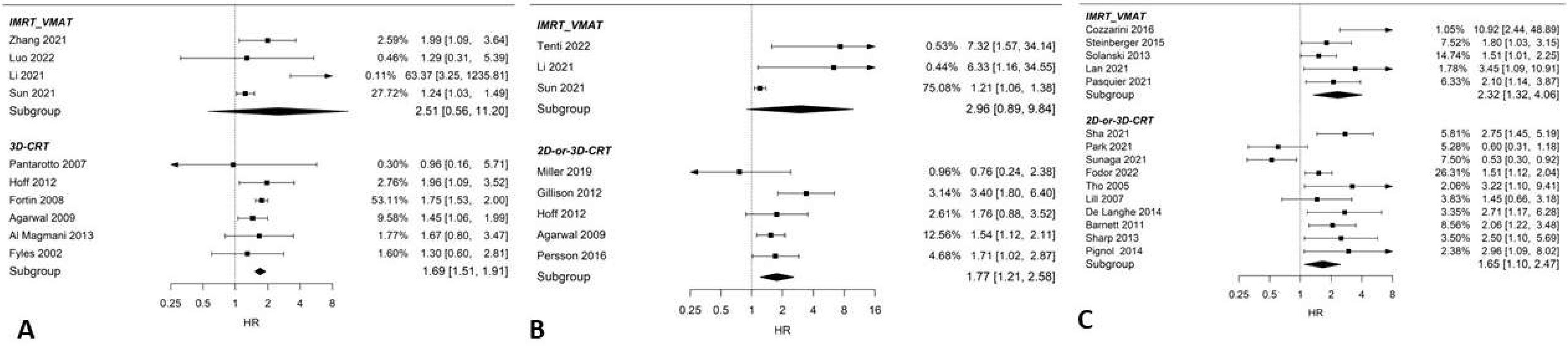

Figure S5. Subgroup analyses based on radiation technique (2D/3D conformal radiation therapy (CRT) vs. intensity modulated radiotherapy (IMRT)/Volumetric Modulated Arc Therapy (VMAT)) for the efficacy (A: locoregional recurrence; B: disease-free survival; Hazard Ratios) and radiation-induced toxicity (C; Odds Ratios) for smokers vs. non-smokers.

Pooled values > 1 indicates worse LRR or DFS or higher risk for radiation-induced toxicity for smokers during immunotherapy while pooled values <1 indicates better LRR or DFS or lower risk for toxicity. Comparison group is non-smokers (former or never).

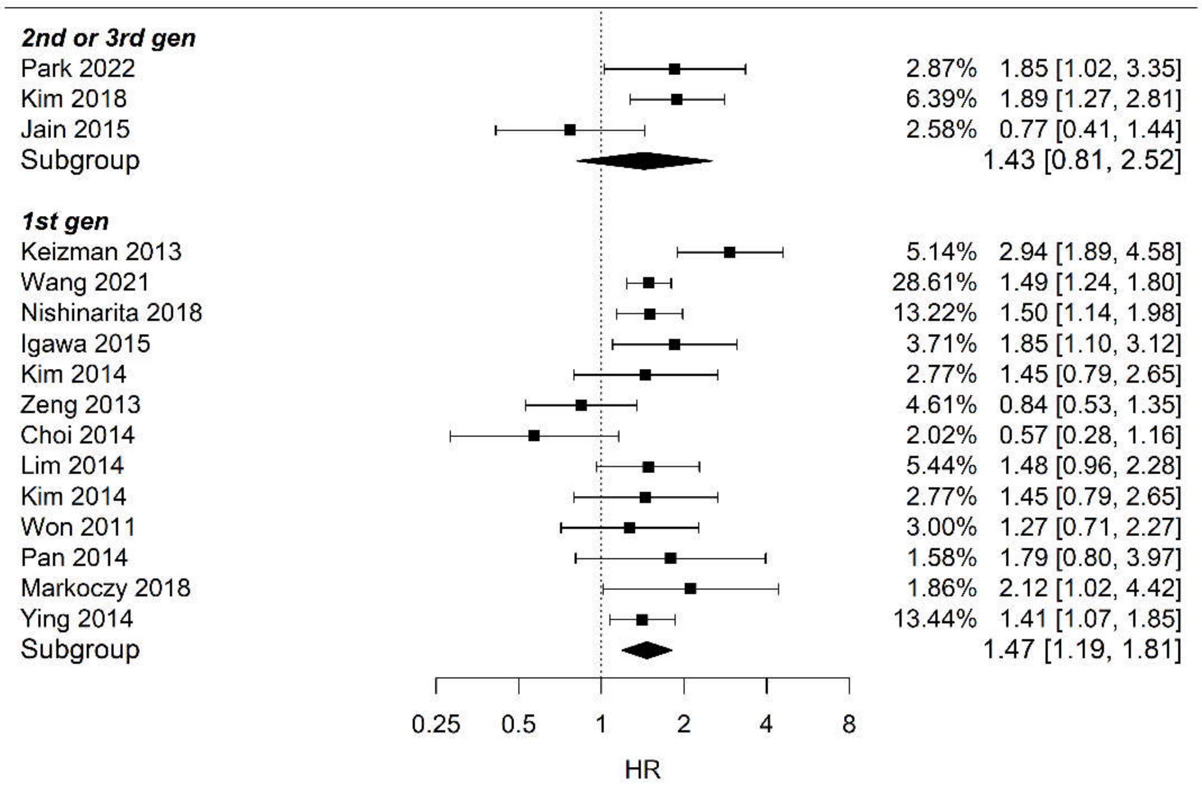

Figure S6 Subgroup analysis based on the generation (1st vs. 2nd or 3rd) of EGFR-tyrosine kinase inhibitors (EGFR-TKIs) and efficacy in terms of progression-free survival (PFS)

HR > 1 indicates worse PFS for smokers during EGFR-TKIs treatment while HR <1 indicates better PFS. Comparison group is non-smoker (former or never)

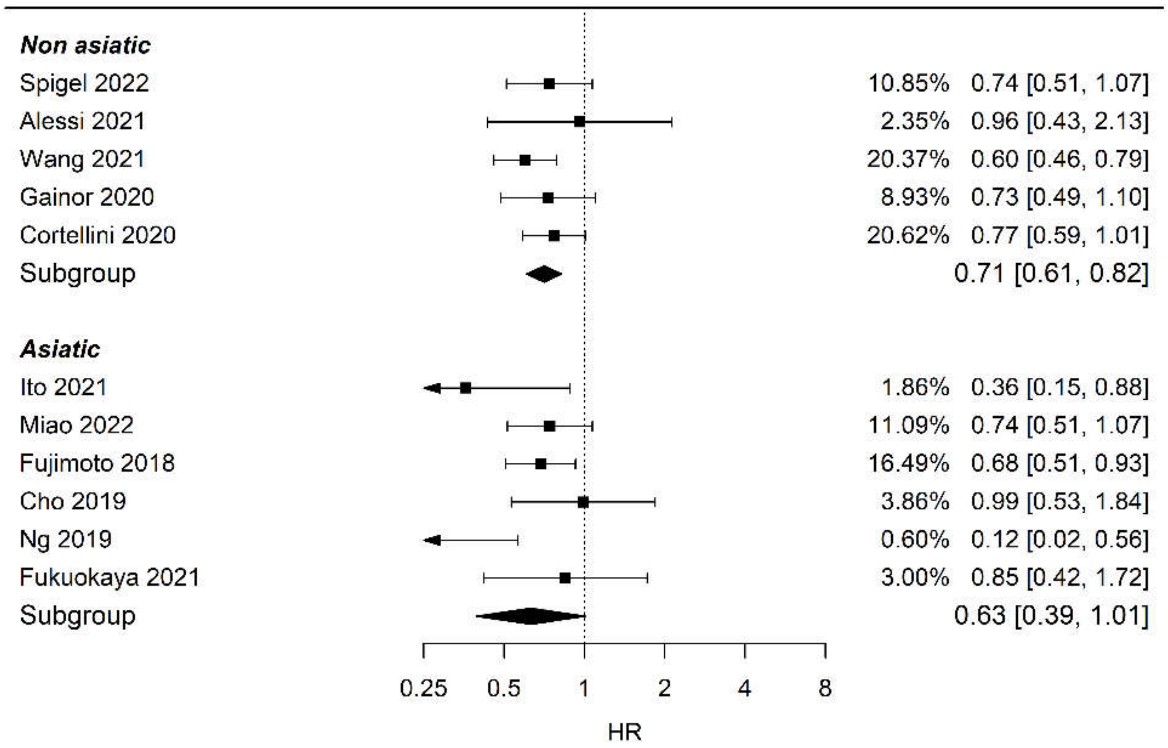

Figure S7 Subgroup analysis based on the race (asiatic vs. non-asiatic population) on smoking during treatment with immunotherapy (checkpoint inhibitors) and efficacy in terms of progression-free survival (PFS)

HR > 1 indicates worse PFS for smokers during immunotherapy while HR <1 indicates better PFS. Comparison group is non-smoker (former or never)

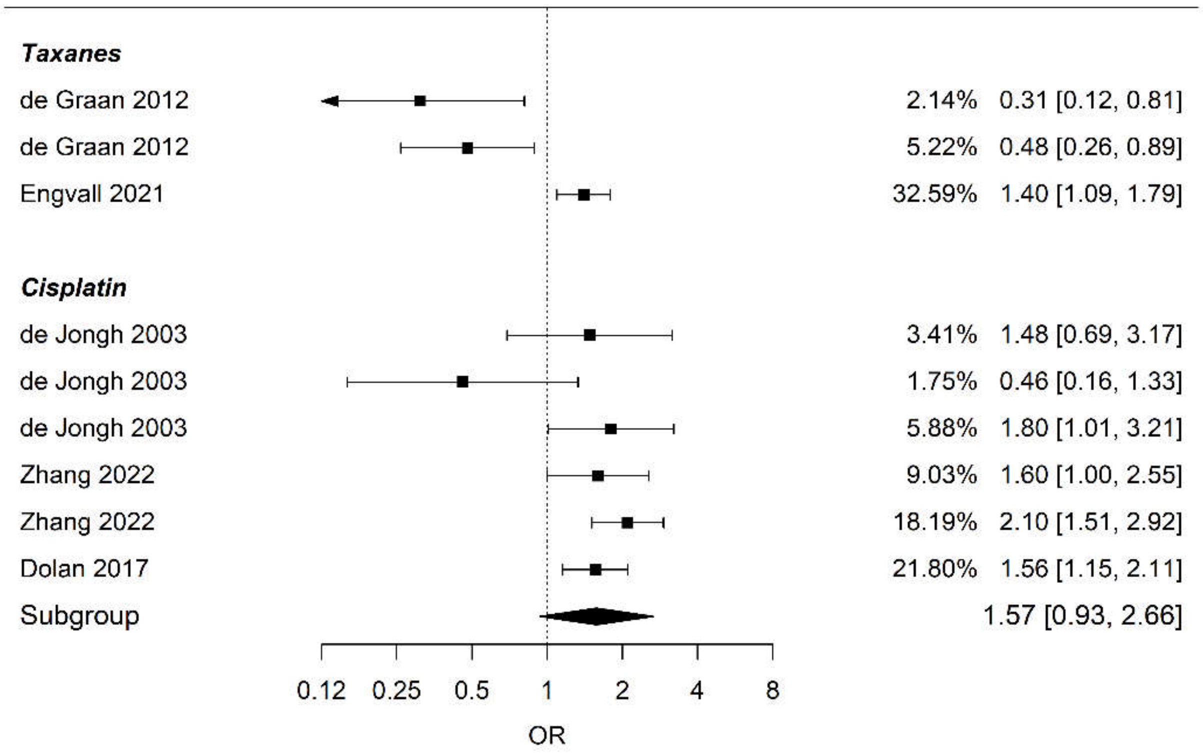

Figure S8 Subgroup analysis based on type of chemotherapeutic agent (taxanes vs. cisplatin) on smoking during chemotherapy and toxicity.

OR > 1 indicates higher risk for toxicity during treatment whereas OR < 1 indicates lower risk. Comparison group is non-smokers (former or never).

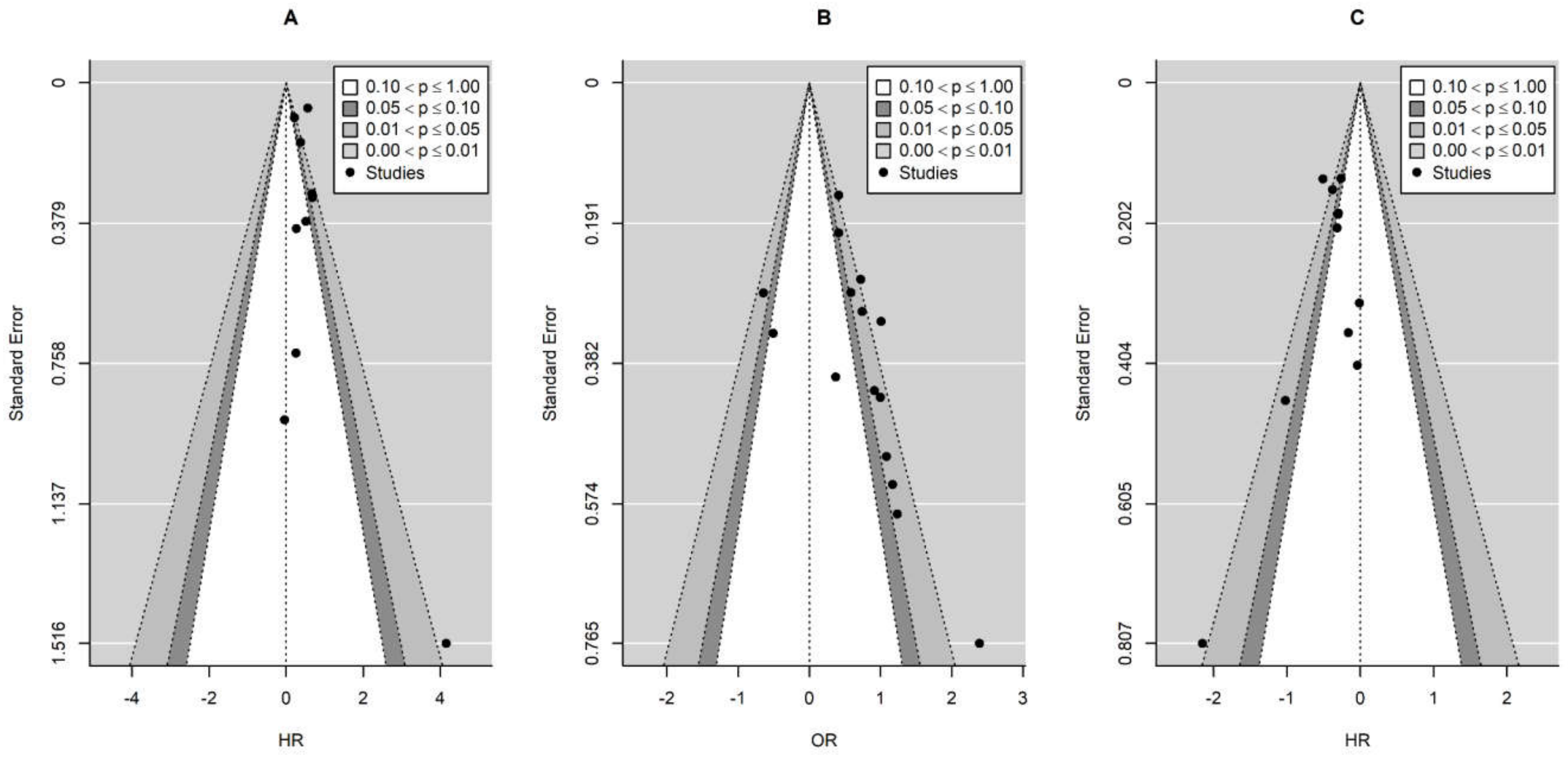

Figure S9 Contour enhanced funnel plots for three meta-analytic models. (A). Locoregional recurrence in patients treated with radiotherapy; (B) Radiation-induced toxicity; (C) progression-free survival in patients treated with immunotherapy. (B) may be suggestive of publication bias with potentially missing studies in areas of non-significance. Corresponding Egger's regression tests for funnel plot asymmetry (A): $z = 1.13$, $p = 0.26$; (B) $z = 2.56$, $p = 0.01$; (C) $z = -1.02$, $p = 0.31$ respectively.
